# Supplementary material for: YAP1 affects the prognosis through the regulation of stemness in endometrial cancer
Source: PeerJ. 2023 Sep 20;11:e15891. doi: 10.7717/peerj.15891 (PMC10517666; doi:10.7717/peerj.15891)
Supplement: Supplemental Information 1 [file peerj-11-15891-s001.docx]

**Supplementary file 1: siRNA Sequences**

| **Item** | | **Sequence** |
| --- | --- | --- |
| **Control** | **Sense** | 5’-UUC UCC GAA CGU GUC ACG UTT-3’ |
|  | **Antisense** | 5’-ACG UGA CAC GUU CGG AGA ATT-3’ |
| **YAP1-siRNA#1** | **Sense** | 5’-GGU GAU ACU AUC AAC CAA ATT-3’ |
|  | **Antisense** | 5’-UUU GGU UGA UAG UAU CAC CTT-3’ |
| **YAP1-siRNA#1** | **Sense** | 5’-CUG CCA CCA AGC UAG AUA ATT-3’ |
|  | **Antisense** | 5’-UUA UCU AGC UUG GUG GCA GTT-3’ |
| **TEAD-1-siRNA** | **Sense** | 5’-GGA CAU UCG UCA GAU UUA UTT -3’ |
|  | **Antisense** | 5’-AUA AAU CUG ACG AAU GUC CTT -3’ |
