# Supplementary material for: YAP1 affects the prognosis through the regulation of stemness in endometrial cancer
Source: PeerJ. 2023 Sep 20;11:e15891. doi: 10.7717/peerj.15891 (PMC10517666; doi:10.7717/peerj.15891)
Supplement: Supplemental Information 2 [file peerj-11-15891-s002.docx]

**Supplementary file 2: Primers**

| **Gene** | **Primer** |
| --- | --- |
| **YAP1** | **(Sense primer)**  5' GCAGAACCGTTTCCCAGACTAC 3'  **(Anti-sense primer)**  5' CAGACTTGGCATCAGCTCCTC 3' |
| **SOX2** | **(Sense primer)**  5' TGGGTTCGGTGGTCAAGTC 3'  **(Anti-sense primer)**  5' GCTCTGGTAGTGCTGGGACA 3' |
| **OCT4** | **(Sense primer)**  5' CAGTATCGAGAACCGAGTGAGA 3'  **(Anti-sense primer)**  5' GATGTGGCTGATCTGCTGC 3' |
| **NANOG** | **(Sense primer)**  5' ACCTATGCCTGTGATTTGTGG 3'  **(Anti-sense primer)**  5' GGTTGTTTGCCTTTGGGAC 3' |
| **CD44** | **(Sense primer)**  5' GCAACCCTACTGATGATGACG 3'  **(Anti-sense primer)**  5' TCTGTCTGTGCTGTCGGTGA 3' |
| **CD133** | **(Sense primer)**  5' TCAAGATACTTCAACGCACAGG 3'  **(Anti-sense primer)**  5' GCACGATGCCACTTTCTCAC 3' |
| **β-actin** | **(Sense primer)**  5' AGAAAATCTGGCACCACACCT 3'  **(Anti-sense primer)**  5' GATAGCACAGCCTGGATAGCA 3' |
| **AMOTL2** | **(Sense primer)**  5' GACACCACTCTCATCCGACATTC 3'  **(Anti-sense primer)**  5' GACCTTGATCACTGCATCCTTCT 3' |
| **ANKRD1** | **(Sense primer)**  5' CTGAAGGCTGCTCTGGAGAATAA 3'  **(Anti-sense primer)**  5' TGGCTGTGGATTCAAGCATATCA 3' |
| **CTGF** | **(Sense primer)**  5' GTTACCAATGACAACGCCTCCT 3'  **(Anti-sense primer)**  5' CGTCGGTACATACTCCACAGAAT 3' |
| **CYR61** | **(Sense primer)**  5' CCTCGCATCCTATACAACCCTTTA 3'  **(Anti-sense primer)**  5' TCTTGGTCTTGCTGCATTTCTTG 3' |
| **TEAD1** | **(Sense primer)**  5' CAAGGTTTGAGAATGGCCGATTT 3'  **(Anti-sense primer)**  5' GTTGTGCTCCGTGTTCACTATTT 3' |
