# Supplementary material for: YAP1 affects the prognosis through the regulation of stemness in endometrial cancer
Source: PeerJ. 2023 Sep 20;11:e15891. doi: 10.7717/peerj.15891 (PMC10517666; doi:10.7717/peerj.15891)
Supplement: Supplemental Information 3 [file peerj-11-15891-s003.docx]

**Supplementary file 3: Protocols of high through-put sequencing and data analysis.**

Total RNA was isolated and purified using TRIzol reagent (Invitrogen, Carlsbad, CA, USA) following the manufacturer's procedure. The RNA amount and purity of each sample was quantified using NanoDrop ND-1000 (NanoDrop, Wilmington, DE, USA), and the RNA integrity was assessed by Bioanalyzer 2100 (Agilent, CA, USA). Poly (A) RNA was purified from 50μg total RNA using Dynabeads Oligo (dT)25-61005 (Thermo Fisher, CA, USA) by two rounds of purification. Then the poly(A) RNA was fragmented into small pieces using Magnesium RNA Fragmentation Module (NEB, cat.e6150, USA) under 86℃ 7min. The cleaved RNA fragments were incubated for 2h at 4℃ with m6A-specific antibody (No. 202003, Synaptic Systems, Germany) in IP buffer (50 mM Tris-HCl, 750 mM NaCl and 0.5% Igepal CA-630). The IP RNA was reverse-transcribed to cDNA by SuperScript™ II Reverse Transcriptase (Invitrogen, cat. 1896649, USA), which was next used to synthesise U-labeled second-stranded DNAs with E. coli DNA polymerase I (NEB, cat.m0209, USA), RNase H (NEB, cat.m0297, USA) and dUTP Solution (Thermo Fisher, cat.R0133, USA）. An A-base was added to the blunt ends of each strand, preparing for ligation to the indexed adapters. Dual-index adapters were ligated to the fragments, and size selection was performed with AMPureXP beads. After the heat-labile UDG enzyme (NEB, cat.m0280, USA) treatment of the U-labeled second-stranded DNAs, the ligated products were amplified with PCR by the following conditions: initial denaturation at 95℃ for 3 min; 8 cycles of denaturation at 98℃ for 15 sec, annealing at 60℃ for 15 sec, and extension at 72℃ for 30 sec; and then final extension at 72℃ for 5 min. At last, we performed paired-end sequencing (PE150) on an Illumina Novaseq™ 6000 platform (LC-Bio Technology CO., Ltd., Hangzhou, China) following the vendor's recommended protocol.

fastp software (https://github.com/OpenGene/fastp) was used to remove the reads that contained adaptor contamination, low quality bases and undetermined bases with default parameter. And sequence quality of IP and Input samples were verified using FastQC (<https://www.bioinformatics.babraham.ac.uk/projects/fastqc/>) and RseQC (<http://rseqc.sourceforge.net/>) . Then we used HISAT2 (<http://daehwankimlab.github.io/hisat2>) to map reads to the reference genome Homo sapiens (Version: v101). And StringTie (<https://ccb.jhu.edu/software/stringtie>) was used to perform expression level for all transcripts and genes from input libraries by calculating FPKM (total exon fragments /mapped reads (millions) × exon length (kB)). The differentially expressed transcripts and genes were selected with log2 (fold change) ≥ 1 or log2 (fold change) ≤ -1 and p value < 0.05 by R package edgeR (<https://bioconductor.org/packages/edgeR>).
