# Supplementary material for: YAP1 affects the prognosis through the regulation of stemness in endometrial cancer
Source: PeerJ. 2023 Sep 20;11:e15891. doi: 10.7717/peerj.15891 (PMC10517666; doi:10.7717/peerj.15891)
Supplement: Supplemental Information 4 [file peerj-11-15891-s004.docx]

**Table Counts of Spheres in ECSC^Ishikawa^**

| Group | Control | siRNA#1 | siRNA#2 |
| --- | --- | --- | --- |
| Counts | 28 | 14 | 22 |
|  | 30 | 12 | 23 |
|  | 35 | 15 | 20 |

**Table Counts of Spheres in ECSC^HEC1-A^**

| Group | Control | siRNA#1 | siRNA#2 |
| --- | --- | --- | --- |
| Counts | 23 | 10 | 13 |
|  | 20 | 14 | 15 |
|  | 18 | 11 | 16 |
