# Supplementary material for: YAP1 affects the prognosis through the regulation of stemness in endometrial cancer
Source: PeerJ. 2023 Sep 20;11:e15891. doi: 10.7717/peerj.15891 (PMC10517666; doi:10.7717/peerj.15891)
Supplement: Supplemental Information 5 [file peerj-11-15891-s005.docx]

The Assessments of YAP1 and CTGF Expression

| Assessments | | YAP1 | |
| --- | --- | --- | --- |
|  |  | High expression | Low expression |
| CTGF | High expression | 6 | 1 |
|  | Low expression | 0 | 13 |

Cohen’Kappa index was 0.886

The Assessments of YAP1 and CYR61 Expression

| Assessments | | YAP1 | |
| --- | --- | --- | --- |
|  |  | High expression | Low expression |
| CYR61 | High expression | 5 | 2 |
|  | Low expression | 1 | 12 |

Cohen’Kappa index was 0.659

The Assessments of YAP1 and ANKRD1 Expression

| Assessments | | YAP1 | |
| --- | --- | --- | --- |
|  |  | High expression | Low expression |
| ANKRD1 | High expression | 5 | 0 |
|  | Low expression | 1 | 14 |

Cohen’Kappa index was 0.875

The Assessments of YAP1 and AMOTL2 Expression

| Assessments | | YAP1 | |
| --- | --- | --- | --- |
|  |  | High expression | Low expression |
| AMOTL2 | High expression | 1 | 13 |
|  | Low expression | 5 | 1 |

Cohen’Kappa index was 0.762

The Assessments of YAP1 and TAZ Expression

| Assessments | | YAP1 | |
| --- | --- | --- | --- |
|  |  | High expression | Low expression |
| TAZ | High expression | 5 | 3 |
|  | Low expression | 1 | 11 |

Cohen’Kappa index was 0.565
