# Supplementary figures and images for: YAP1 affects the prognosis through the regulation of stemness in endometrial cancer
Source: PeerJ. 2023 Sep 20;11:e15891. doi: 10.7717/peerj.15891 (PMC10517666; doi:10.7717/peerj.15891)

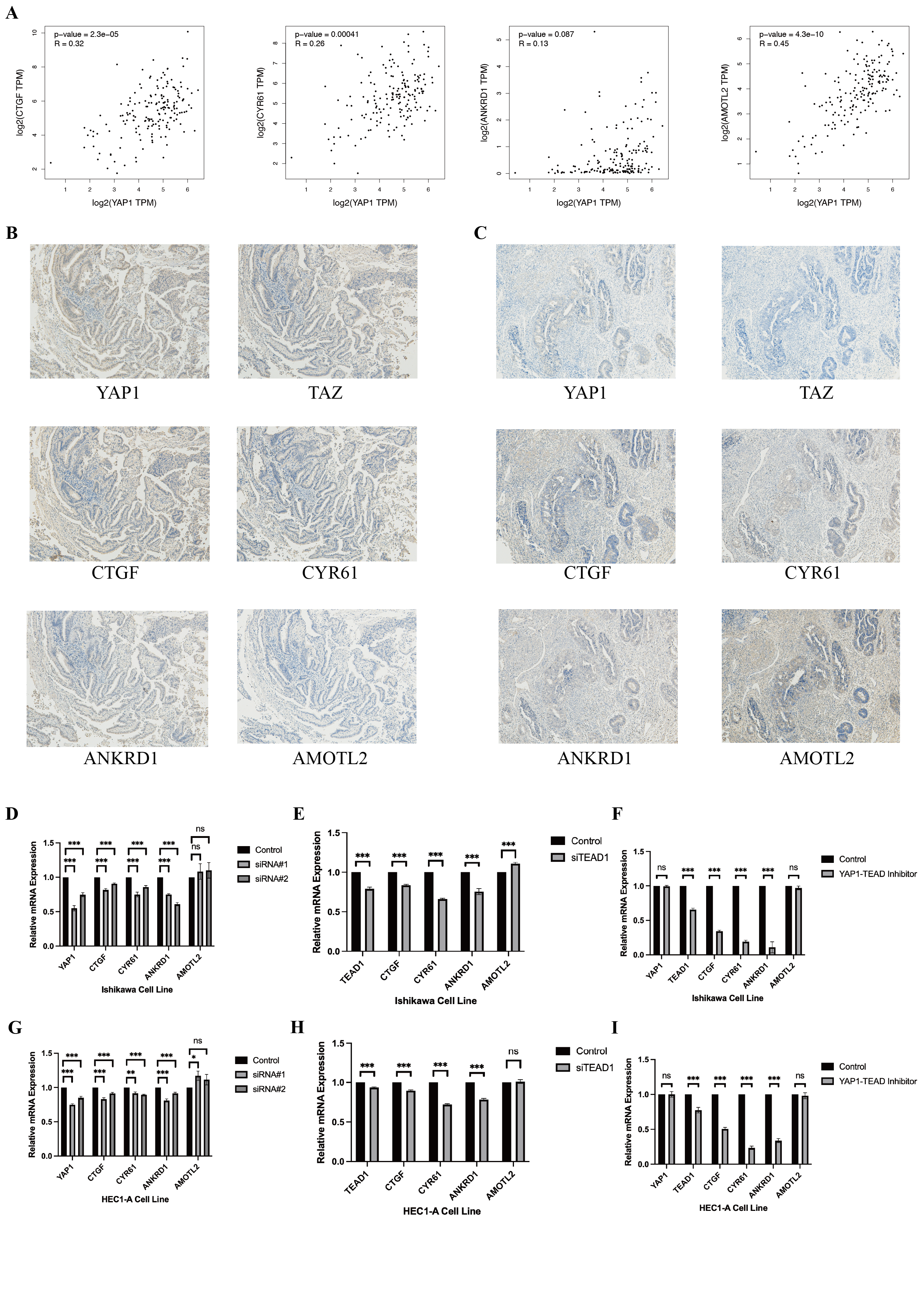

Supplement: Supplemental Information 6 [file peerj-11-15891-s006.png]

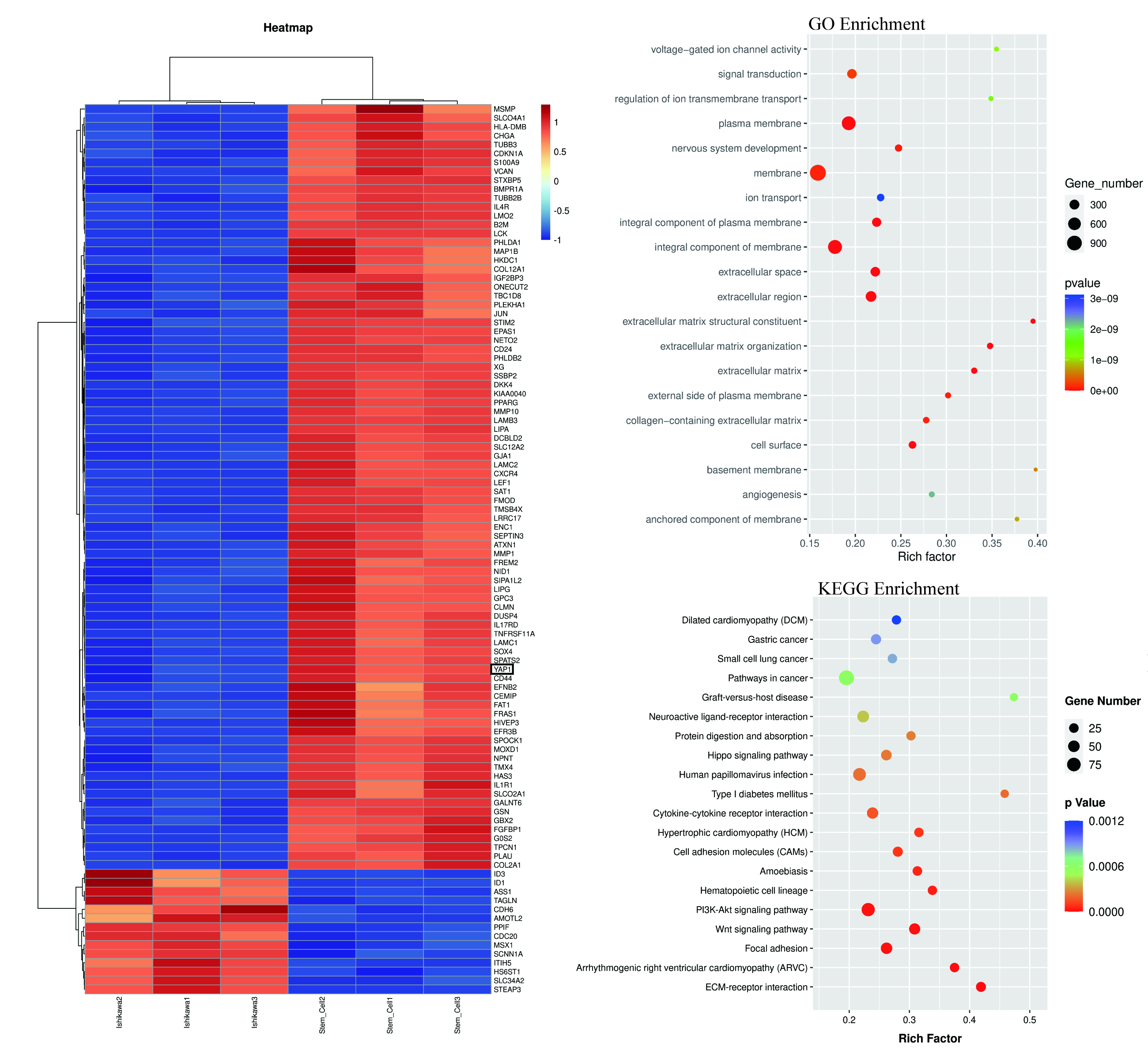

Supplement: Supplemental Information 7 — The names of the genes are shown on the right. Fourteen of the top 100 genes are down-regulated in stem cells (below) and the rest are up-regulated in stem cells (above). [file peerj-11-15891-s007.tif]

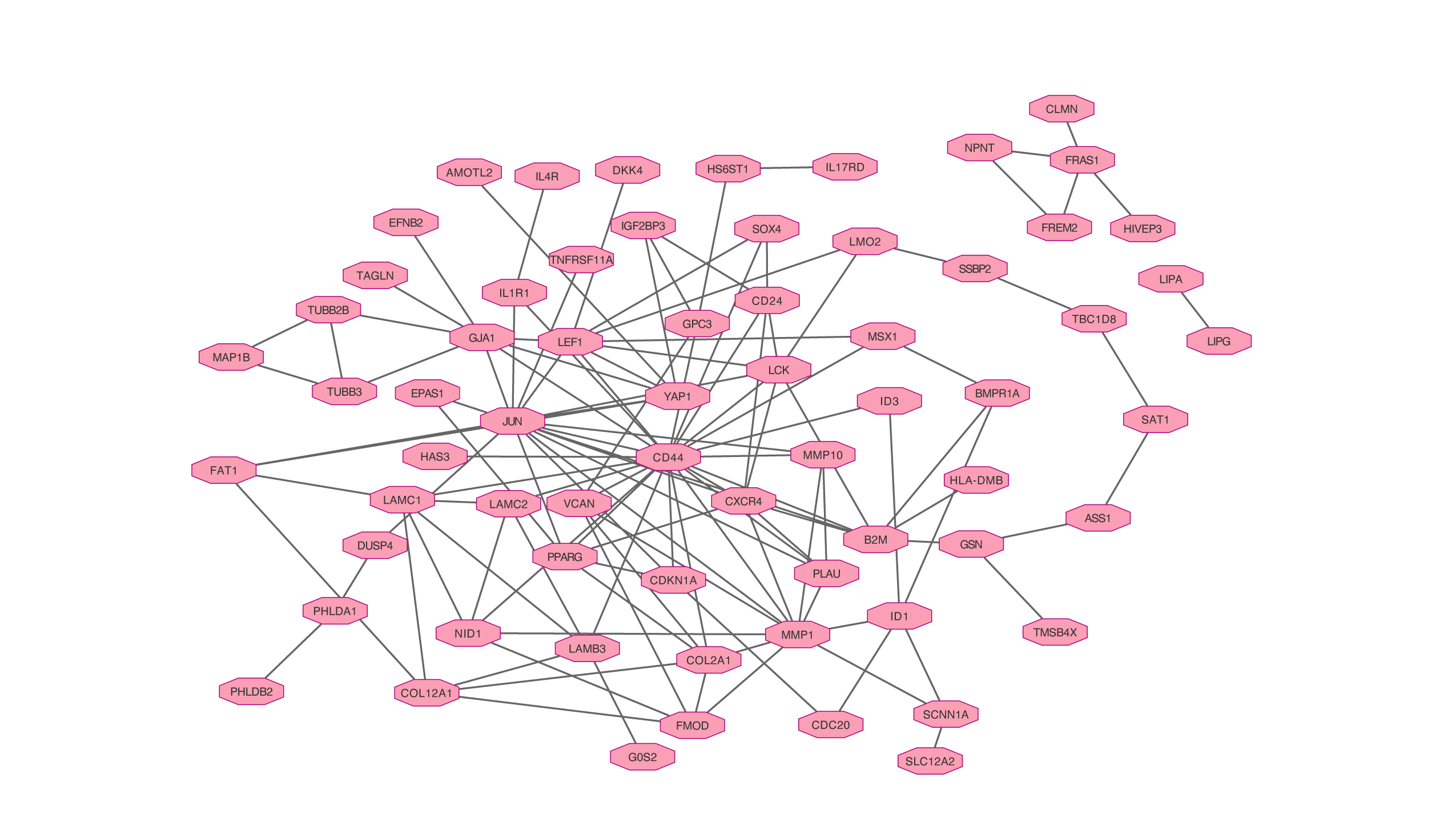

Supplement: Supplemental Information 8 [file peerj-11-15891-s008.tif]

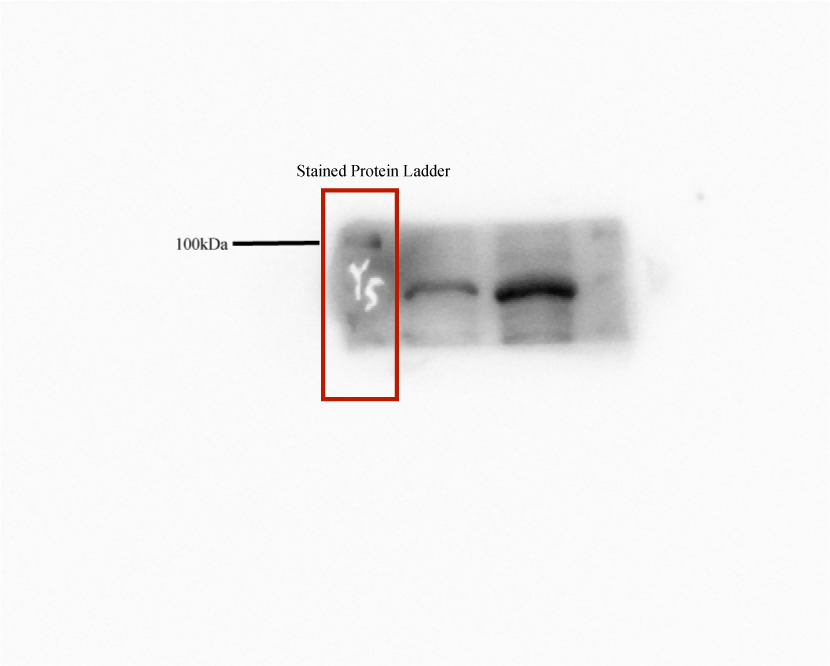

Supplement: Supplemental Information 9 [file peerj-11-15891-s009.zip › Figure2 WB 0122/Figure2G YAP1-1.tif]

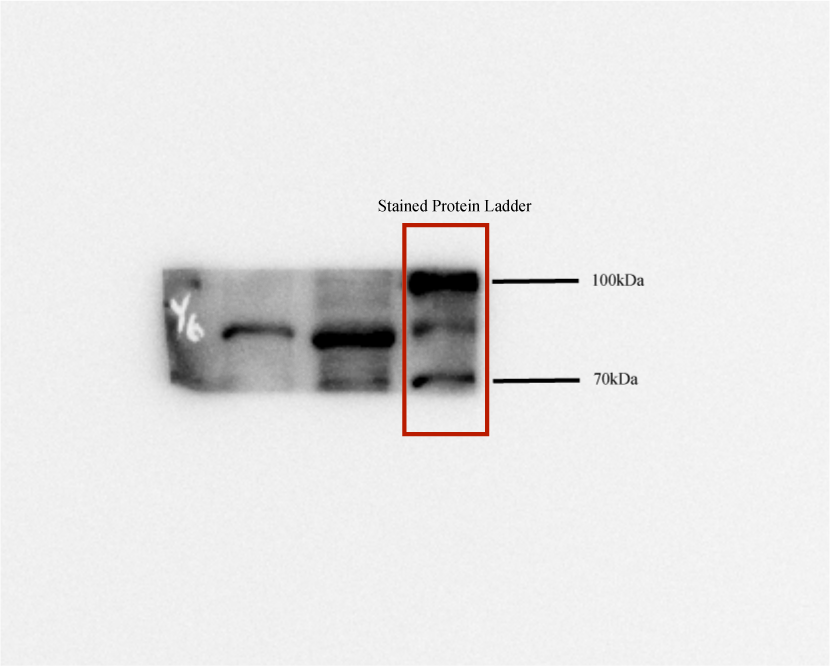

Supplement: Supplemental Information 9 [file peerj-11-15891-s009.zip › Figure2 WB 0122/Figure2G YAP1-2.tif]

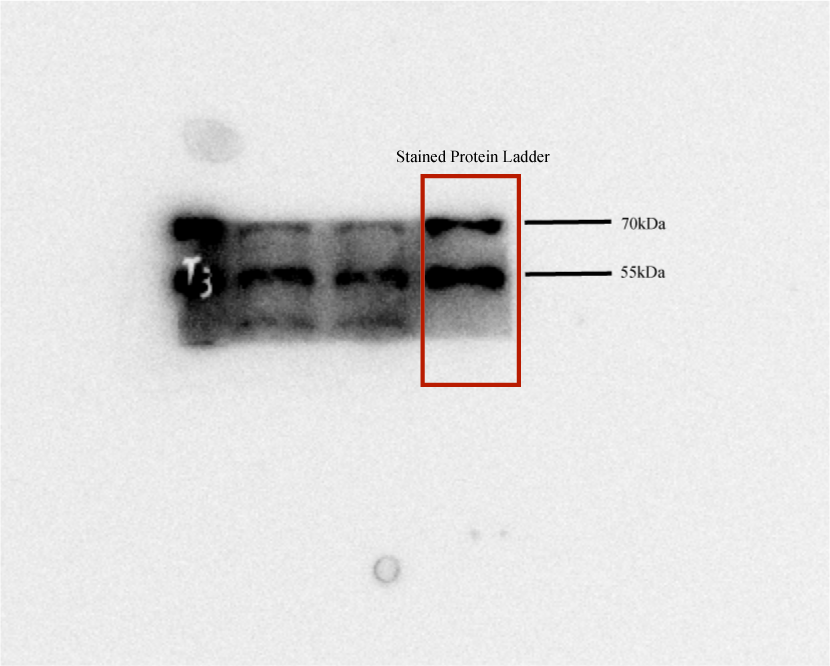

Supplement: Supplemental Information 9 [file peerj-11-15891-s009.zip › Figure2 WB 0122/Figure2E Tubulin.tif]

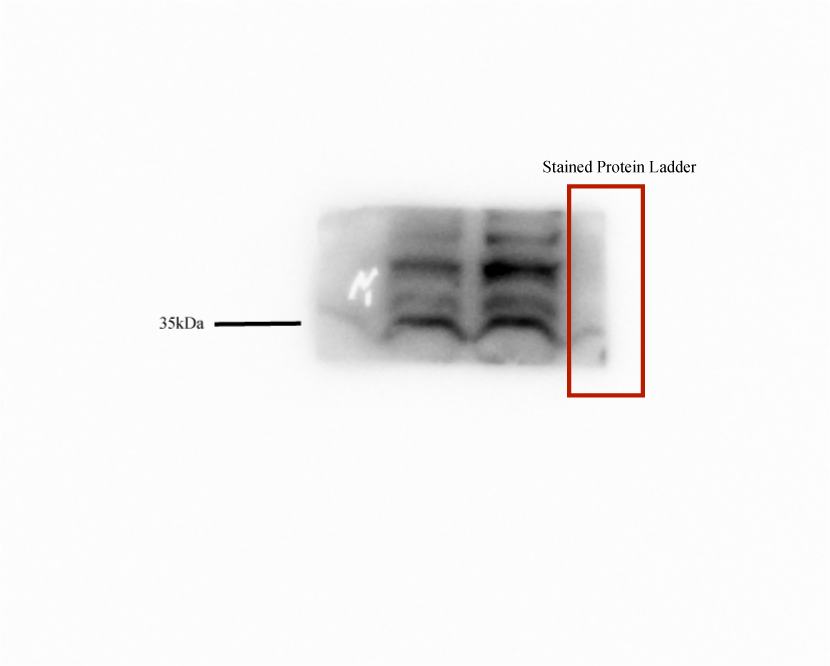

Supplement: Supplemental Information 9 [file peerj-11-15891-s009.zip › Figure2 WB 0122/Figure2D NANOG.tif]

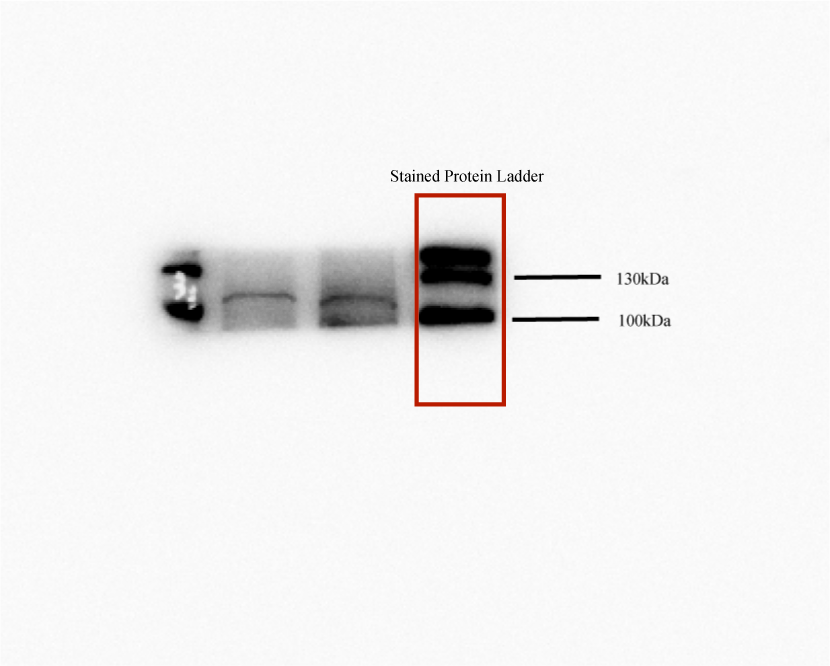

Supplement: Supplemental Information 9 [file peerj-11-15891-s009.zip › Figure2 WB 0122/Figure2E CD133.tif]

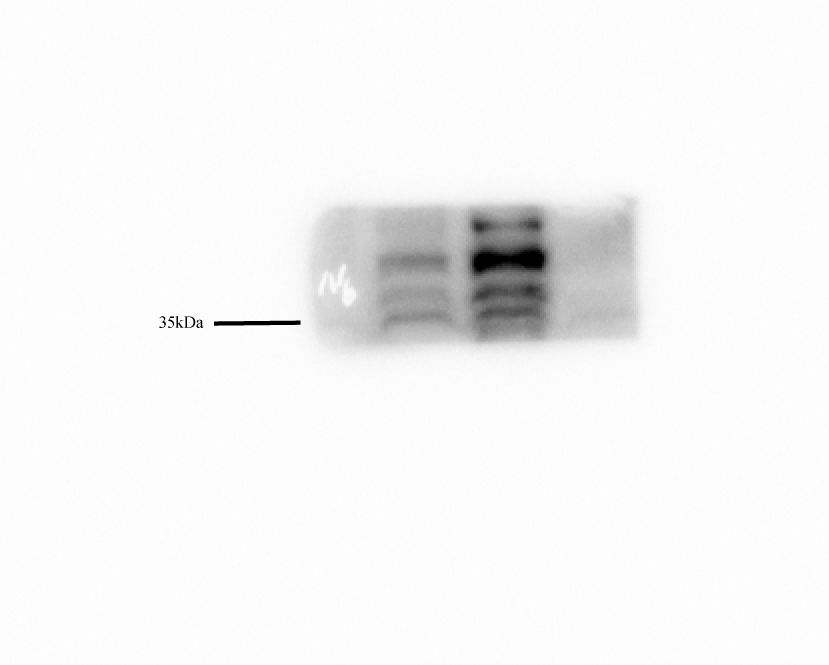

Supplement: Supplemental Information 9 [file peerj-11-15891-s009.zip › Figure2 WB 0122/Figure2E NANOG.tif]

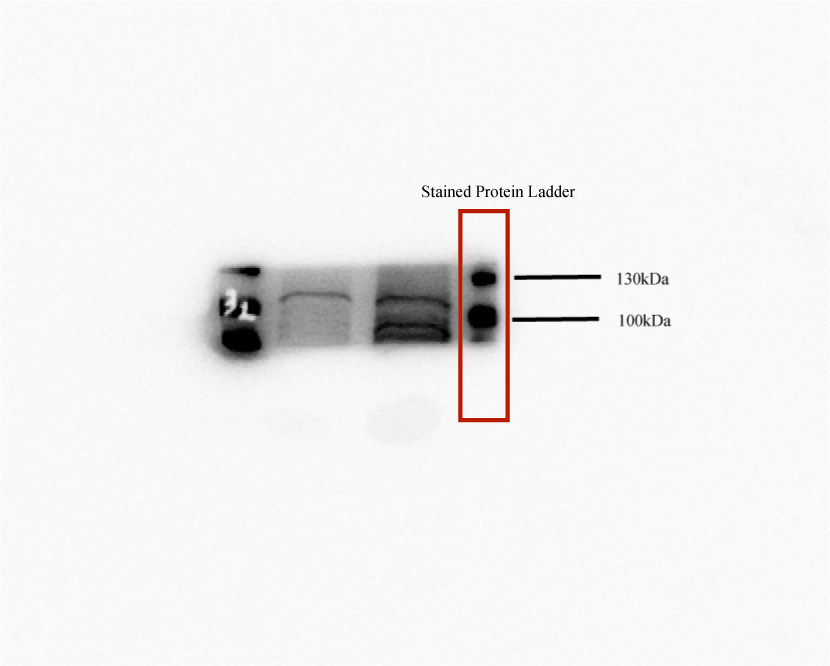

Supplement: Supplemental Information 9 [file peerj-11-15891-s009.zip › Figure2 WB 0122/Figure2D CD133.tif]

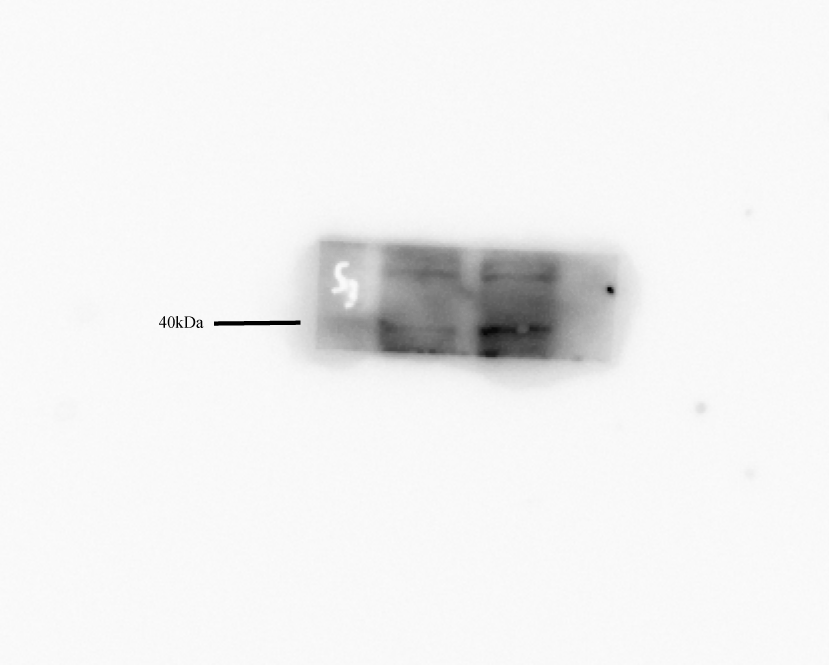

Supplement: Supplemental Information 9 [file peerj-11-15891-s009.zip › Figure2 WB 0122/Figure2E SOX2.tif]

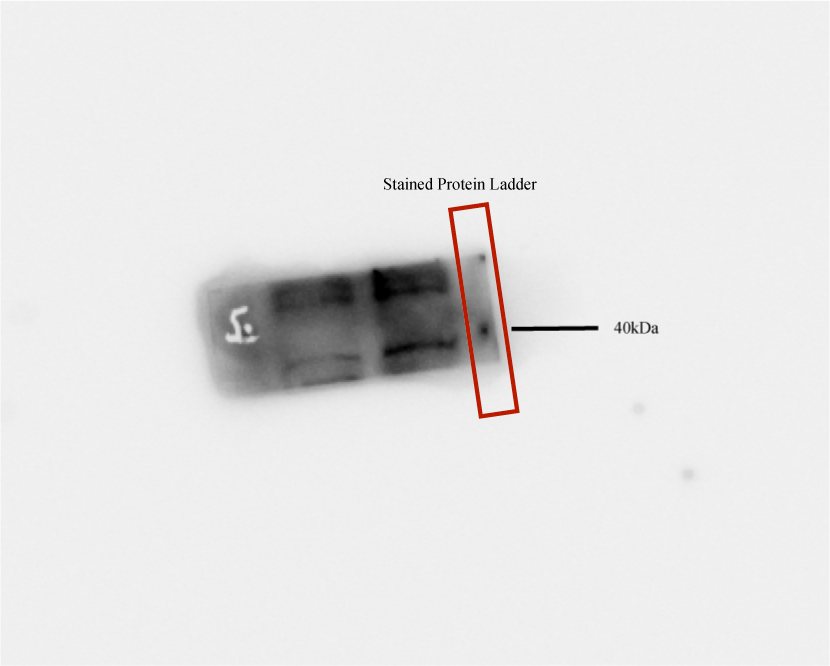

Supplement: Supplemental Information 9 [file peerj-11-15891-s009.zip › Figure2 WB 0122/Figure2D SOX2.tif]

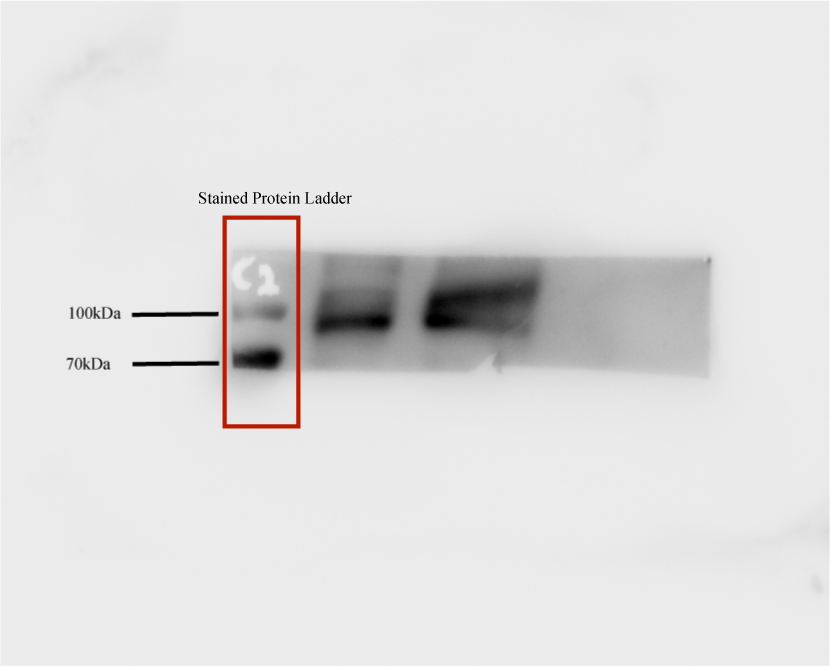

Supplement: Supplemental Information 9 [file peerj-11-15891-s009.zip › Figure2 WB 0122/Figure2D CD44.tif]

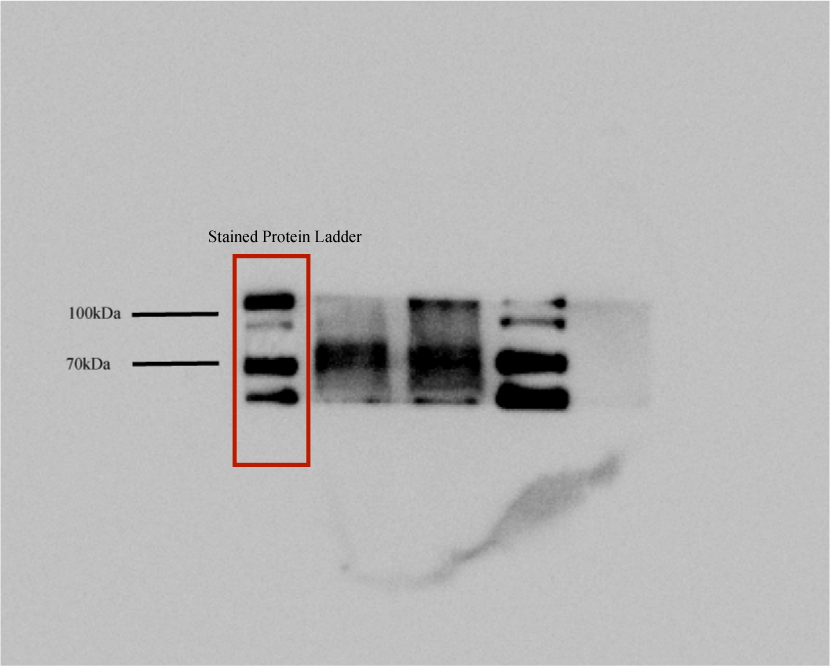

Supplement: Supplemental Information 9 [file peerj-11-15891-s009.zip › Figure2 WB 0122/Figure2E CD44.tif]

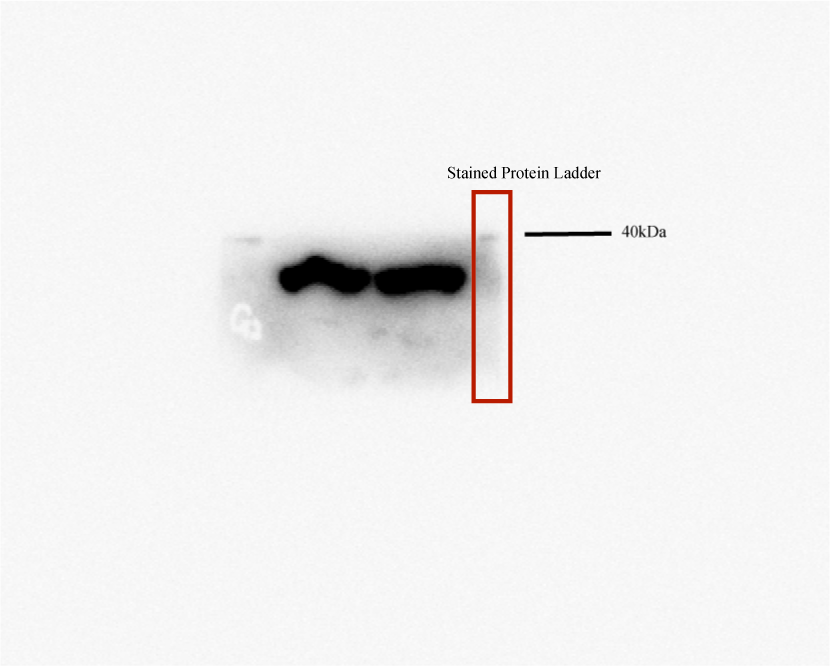

Supplement: Supplemental Information 9 [file peerj-11-15891-s009.zip › Figure2 WB 0122/Figure2G GAPDH2.tif]

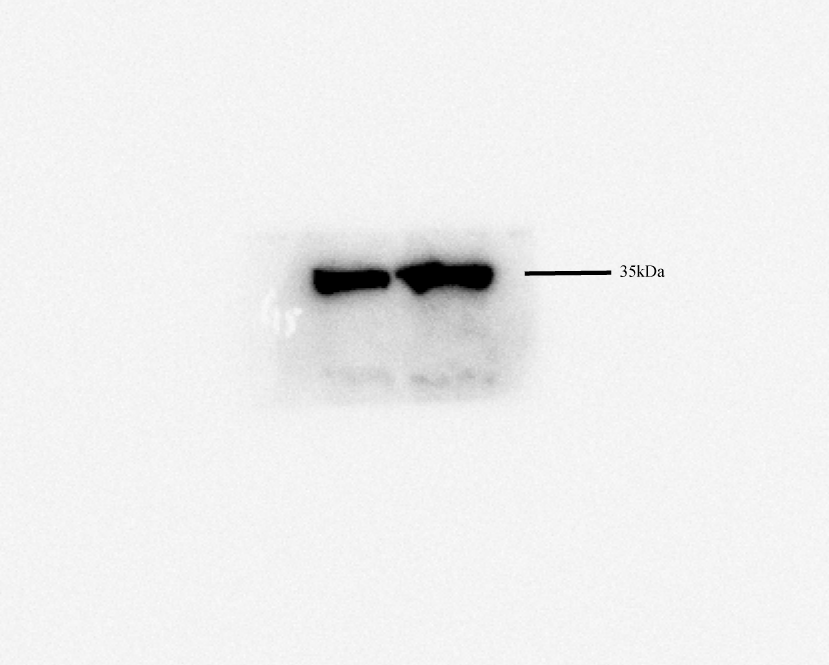

Supplement: Supplemental Information 9 [file peerj-11-15891-s009.zip › Figure2 WB 0122/Figure2E GAPDH.tif]

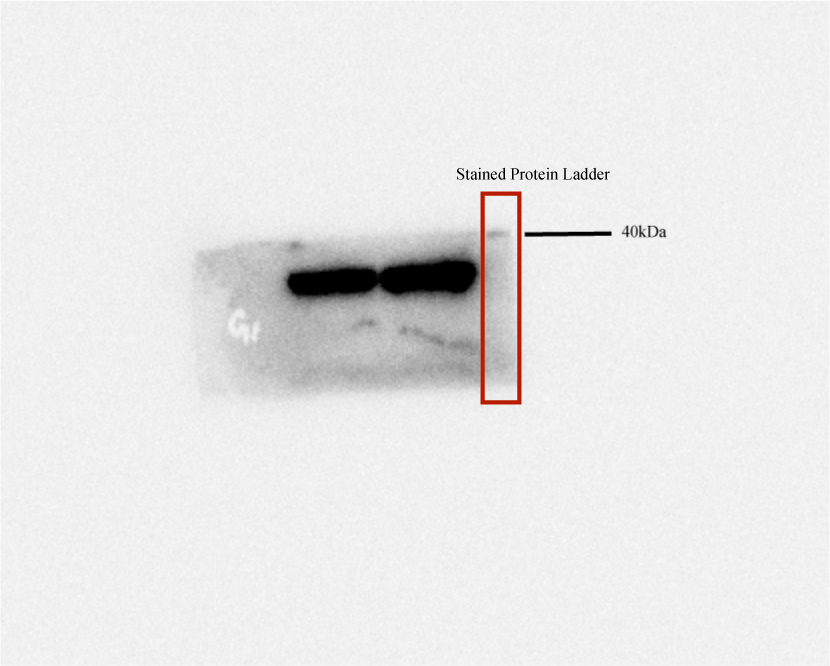

Supplement: Supplemental Information 9 [file peerj-11-15891-s009.zip › Figure2 WB 0122/Figure2G GAPDH1.tif]

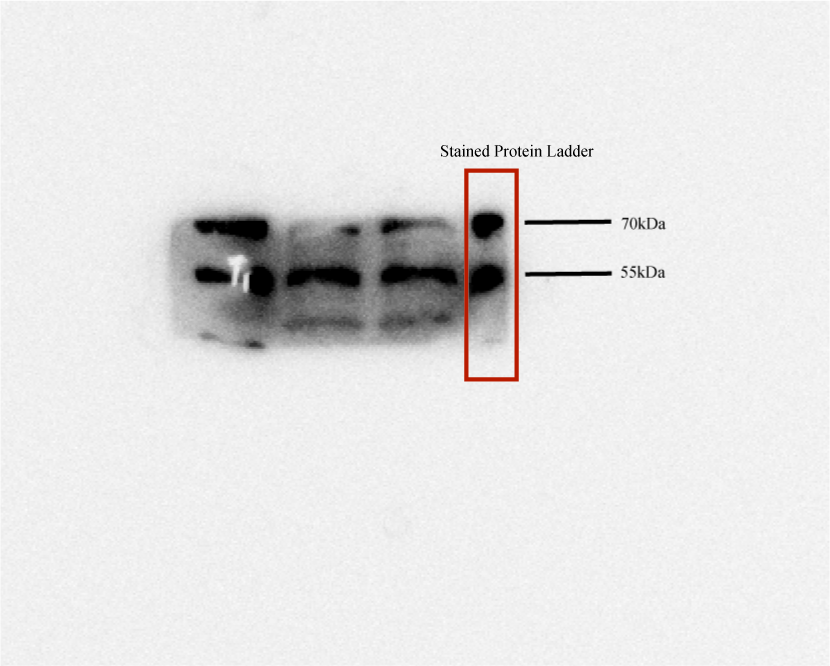

Supplement: Supplemental Information 9 [file peerj-11-15891-s009.zip › Figure2 WB 0122/Figure2D Tubulin.tif]

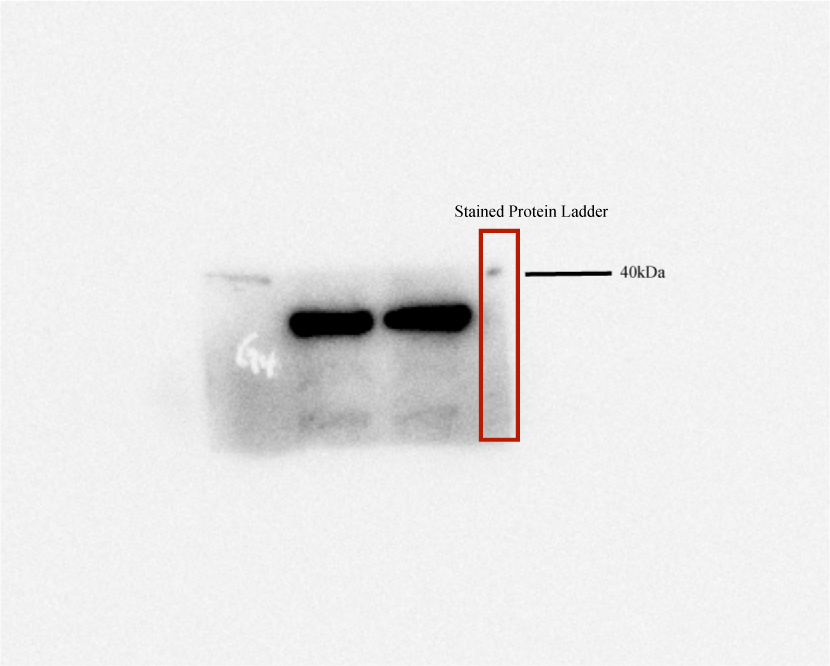

Supplement: Supplemental Information 9 [file peerj-11-15891-s009.zip › Figure2 WB 0122/Figure2D GAPDH.tif]

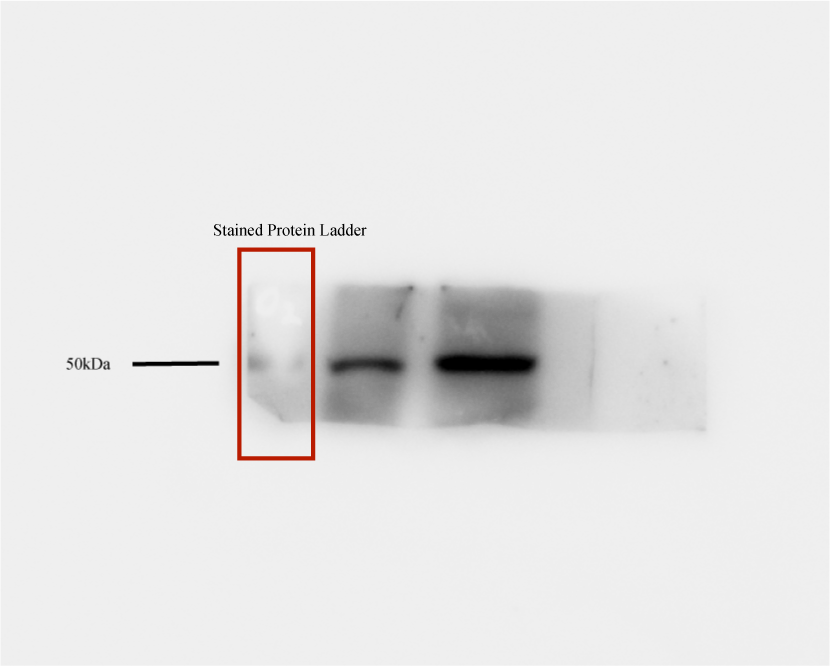

Supplement: Supplemental Information 9 [file peerj-11-15891-s009.zip › Figure2 WB 0122/Figure2D OCT4.tif]

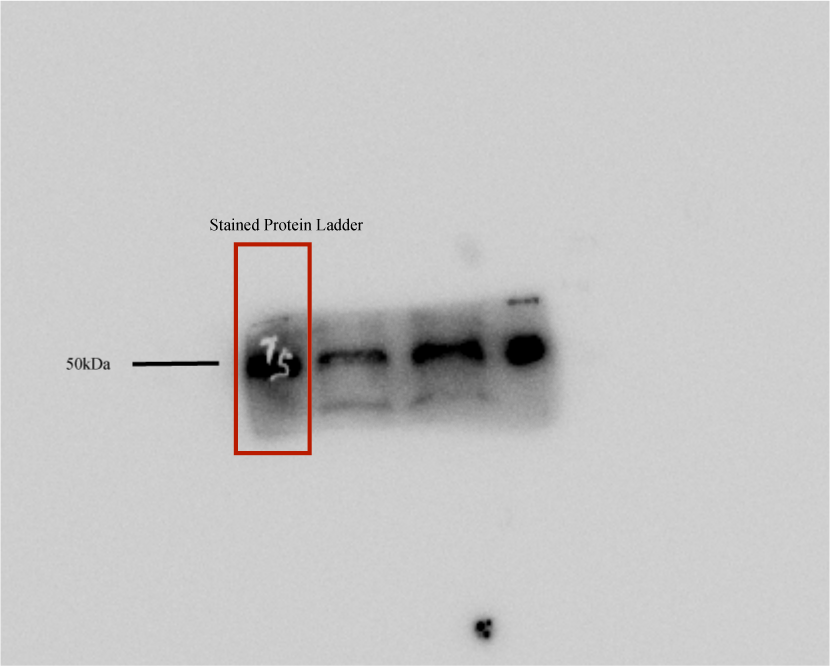

Supplement: Supplemental Information 9 [file peerj-11-15891-s009.zip › Figure2 WB 0122/Figure2E OCT4.tif]

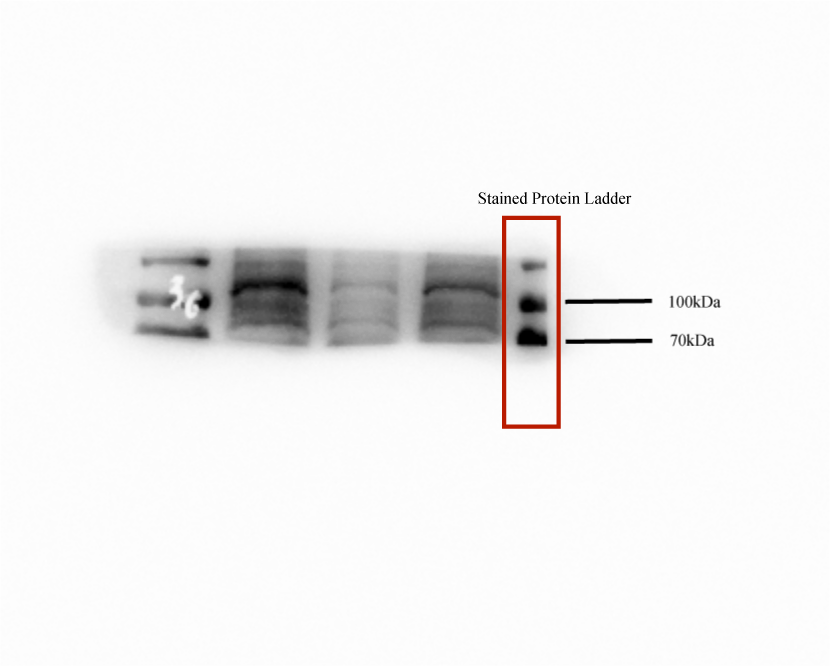

Supplement: Supplemental Information 10 [file peerj-11-15891-s010.zip › Figure3 WB 0122/Figure3J CD133.tif]

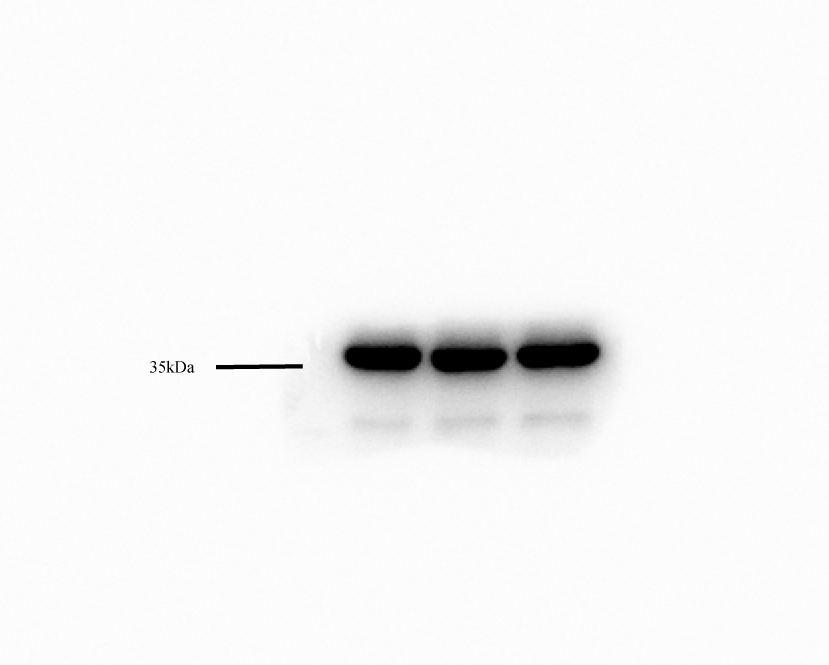

Supplement: Supplemental Information 10 [file peerj-11-15891-s010.zip › Figure3 WB 0122/Figure3H GAPDH.tif]

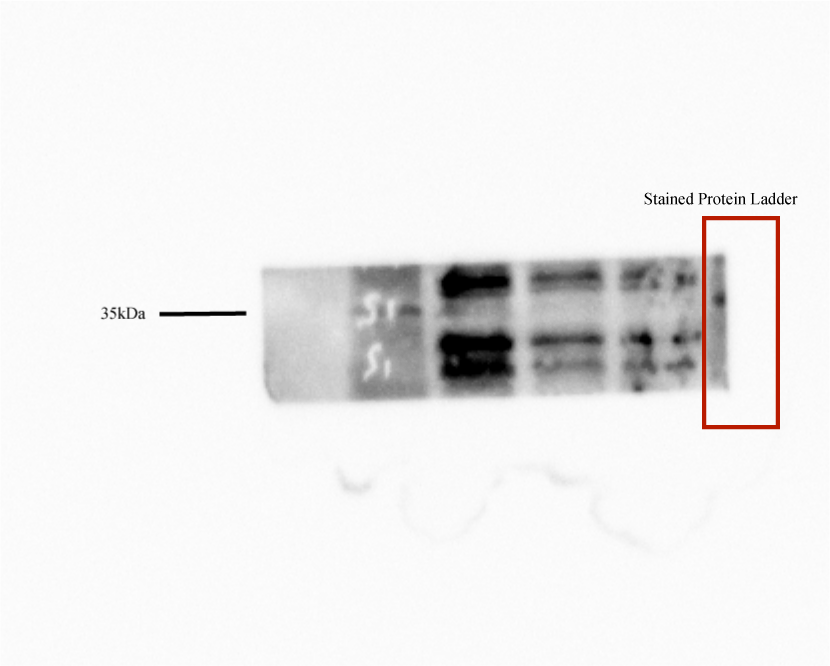

Supplement: Supplemental Information 10 [file peerj-11-15891-s010.zip › Figure3 WB 0122/Figure3H SOX2.tif]

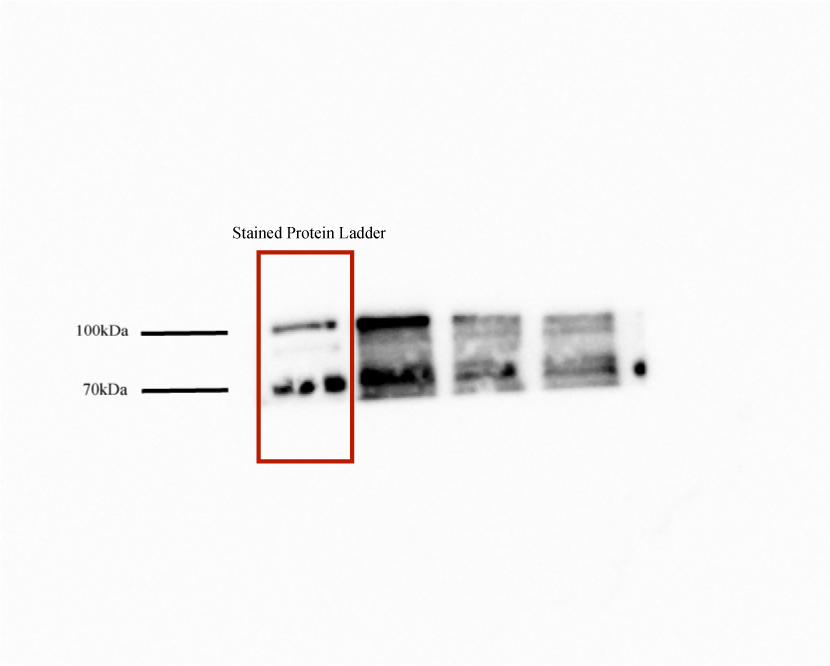

Supplement: Supplemental Information 10 [file peerj-11-15891-s010.zip › Figure3 WB 0122/Figure3H CD44.tif]

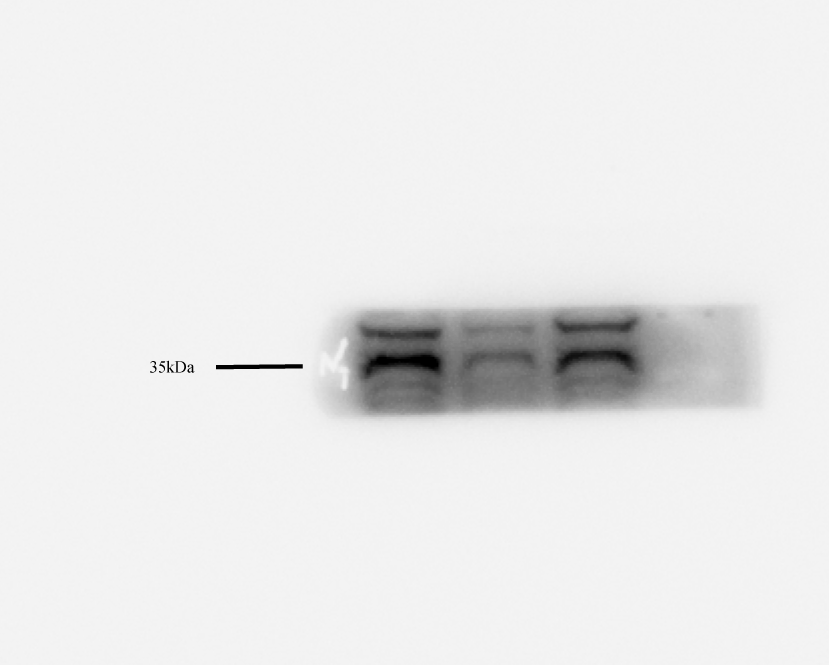

Supplement: Supplemental Information 10 [file peerj-11-15891-s010.zip › Figure3 WB 0122/Figure3J NANOG.tif]

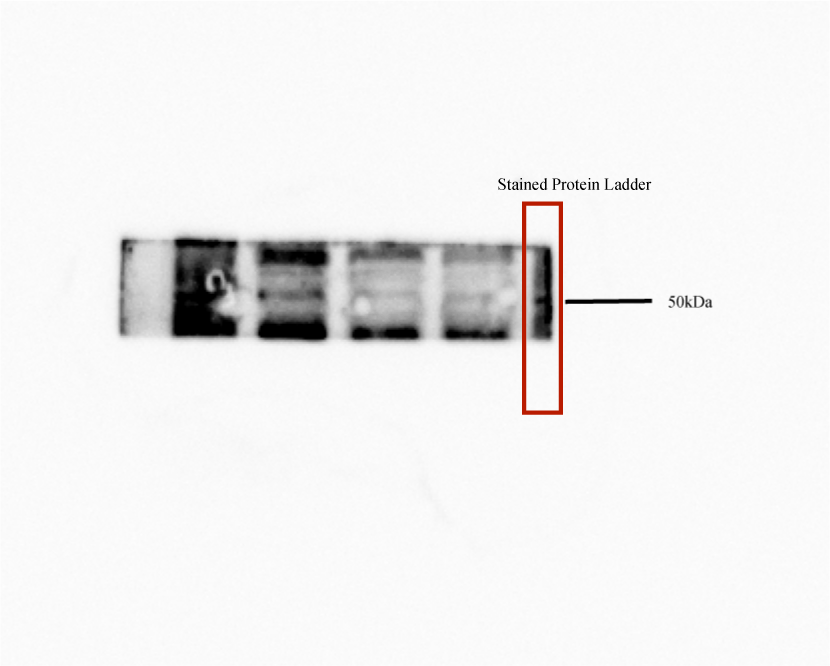

Supplement: Supplemental Information 10 [file peerj-11-15891-s010.zip › Figure3 WB 0122/Figure3J OCT4.tif]

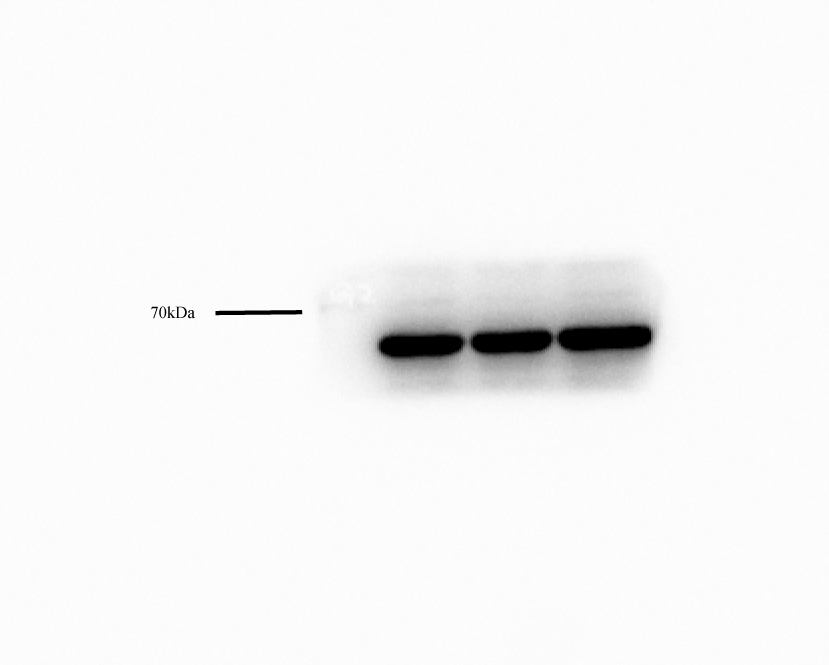

Supplement: Supplemental Information 10 [file peerj-11-15891-s010.zip › Figure3 WB 0122/Figure3J Tubulin.tif]

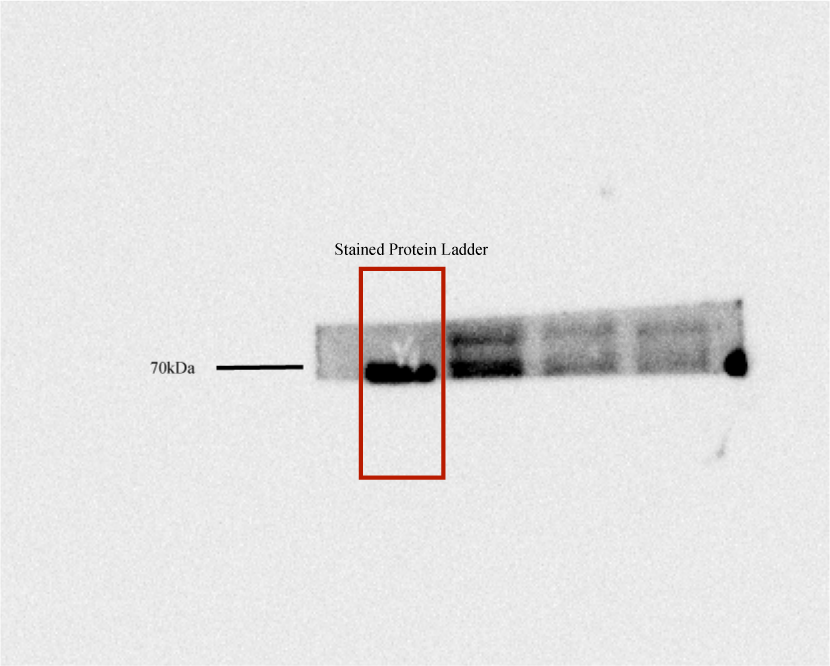

Supplement: Supplemental Information 10 [file peerj-11-15891-s010.zip › Figure3 WB 0122/Figure3H YAP1.tif]

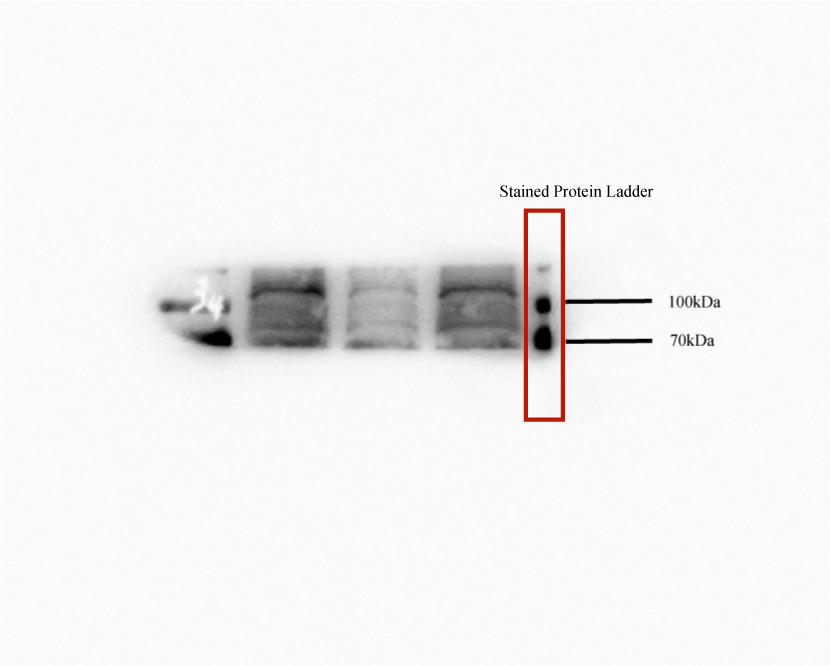

Supplement: Supplemental Information 10 [file peerj-11-15891-s010.zip › Figure3 WB 0122/Figure3H CD133.tif]

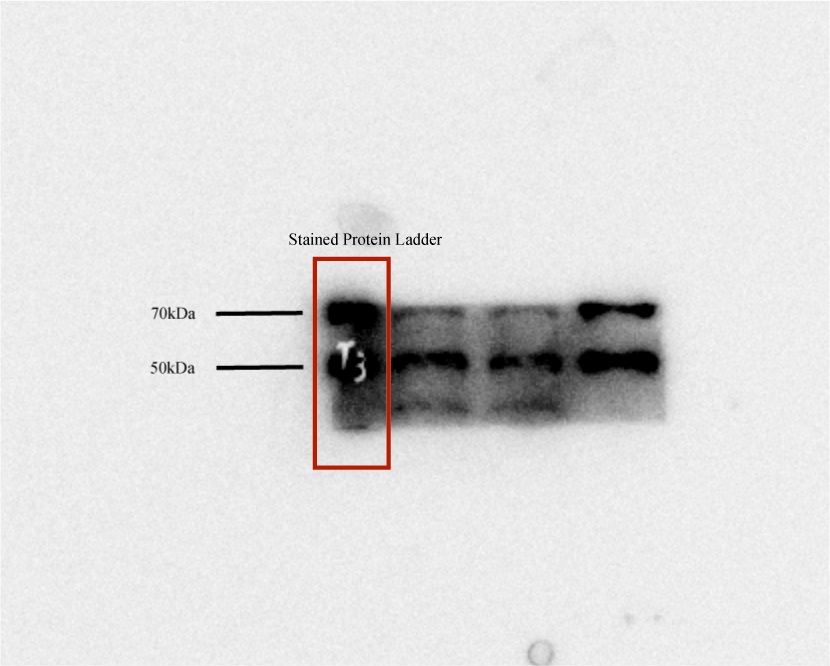

Supplement: Supplemental Information 10 [file peerj-11-15891-s010.zip › Figure3 WB 0122/Figure3H Tubulin.tif]

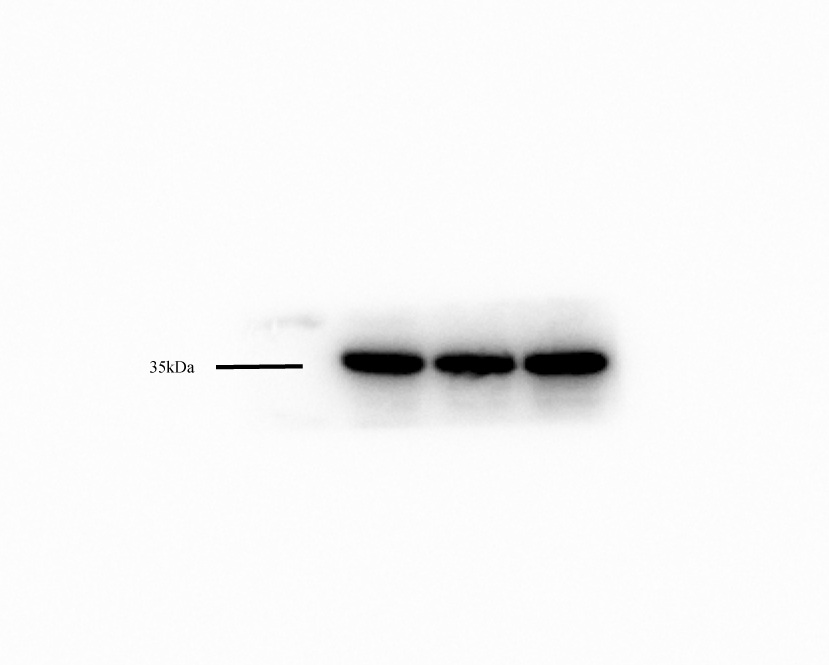

Supplement: Supplemental Information 10 [file peerj-11-15891-s010.zip › Figure3 WB 0122/Figure3J GAPDH.tif]

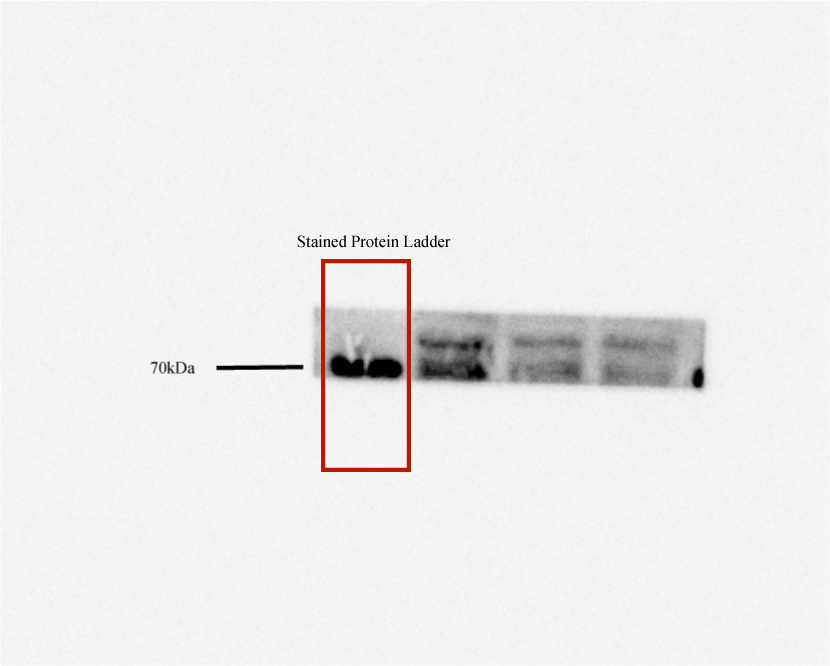

Supplement: Supplemental Information 10 [file peerj-11-15891-s010.zip › Figure3 WB 0122/Figure3J YAP1.tif]

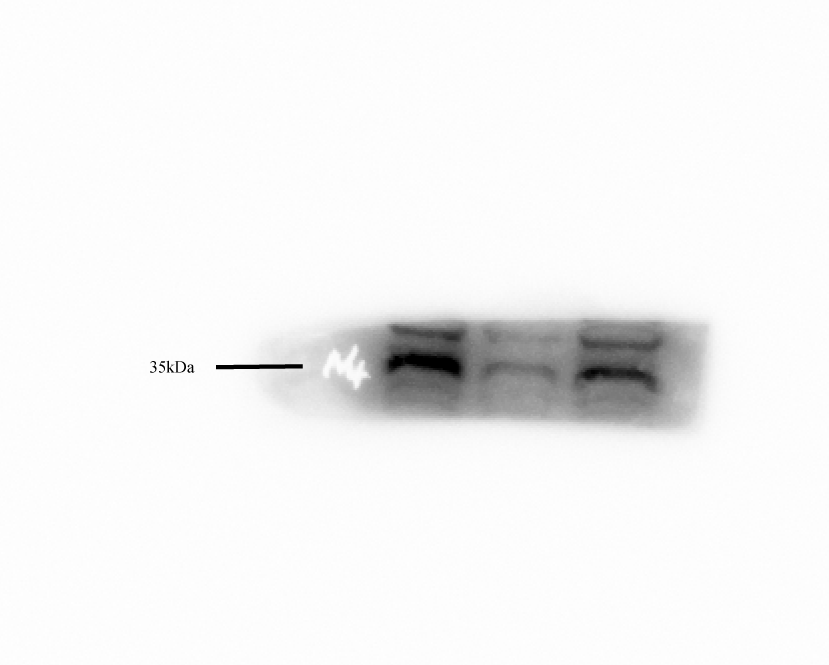

Supplement: Supplemental Information 10 [file peerj-11-15891-s010.zip › Figure3 WB 0122/Figure3H NANOG.tif]

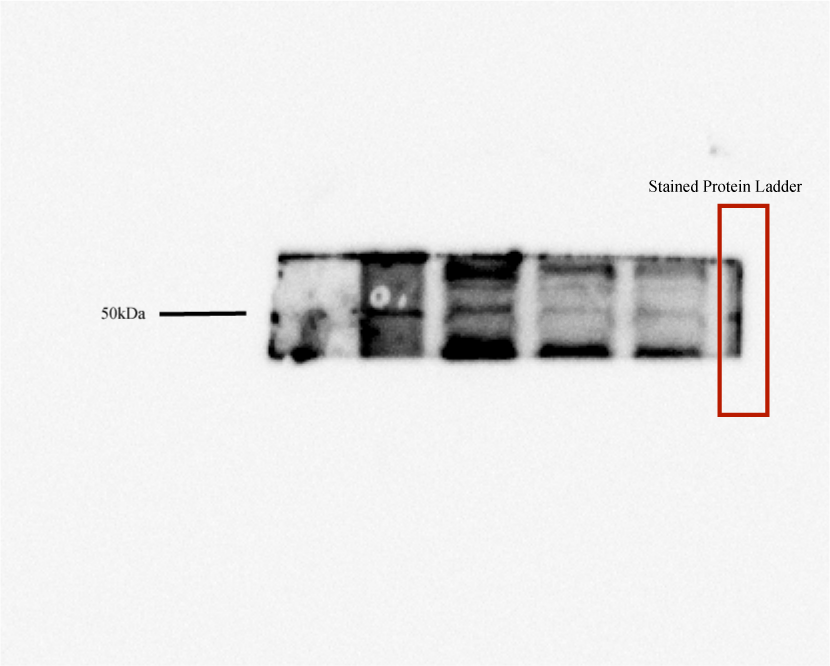

Supplement: Supplemental Information 10 [file peerj-11-15891-s010.zip › Figure3 WB 0122/Figure3H OCT4.tif]

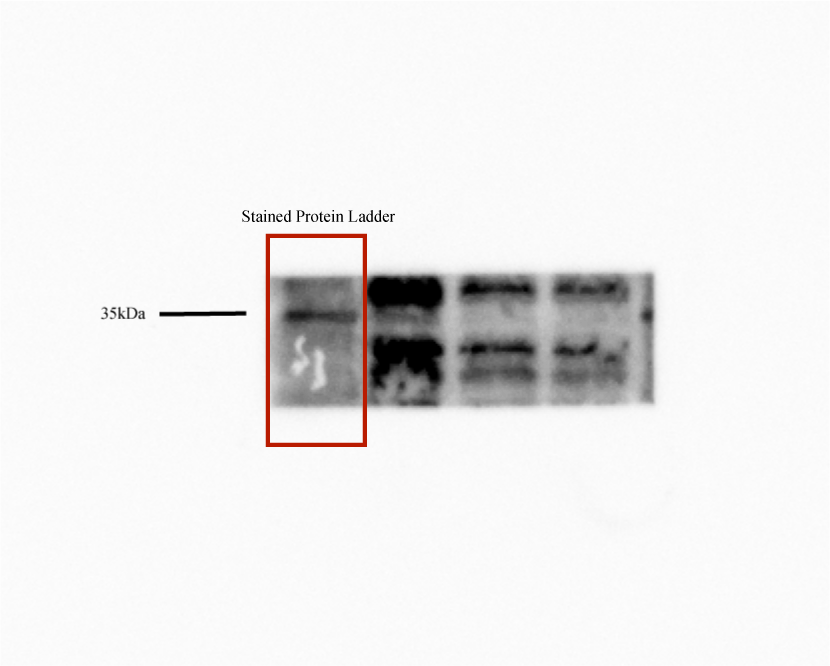

Supplement: Supplemental Information 10 [file peerj-11-15891-s010.zip › Figure3 WB 0122/Figure3J SOX2.tif]

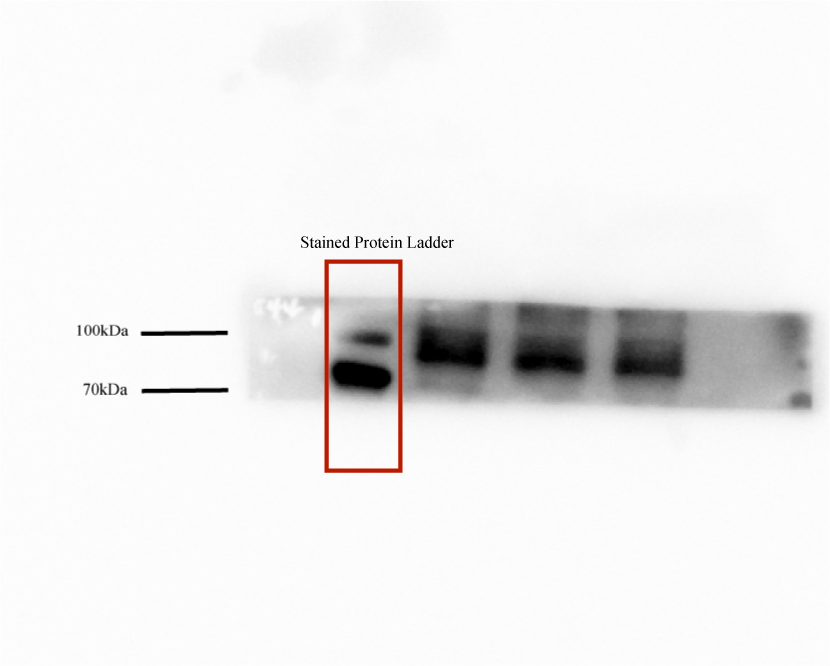

Supplement: Supplemental Information 10 [file peerj-11-15891-s010.zip › Figure3 WB 0122/Figure3J CD44.tif]
